# No evidence of autoimmunity to human OX_1_ or OX_2_ orexin receptors in Pandemrix-vaccinated narcoleptic children

**DOI:** 10.1016/j.jtauto.2020.100055

**Published:** 2020-05-01

**Authors:** Krister Melén, Pinja Jalkanen, Jyrki P. Kukkonen, Markku Partinen, Hanna Nohynek, Arja Vuorela, Outi Vaarala, Tobias L. Freitag, Seppo Meri, Ilkka Julkunen

**Affiliations:** a Institute of Biomedicine, University of Turku, Kiinamyllynkatu 10, 20520, Turku, Finland; b Expert Microbiology Unit, Finnish Institute for Health and Welfare, Mannerheimintie 166, 00300, Helsinki, Finland; c Department of Physiology and Department of Pharmacology, Institute of Biomedicine, Faculty of Medicine and Biochemistry and Cell Biology, Department of Veterinary Biosciences, Faculty of Veterinary Medicine, University of Helsinki, Helsinki, Finland; d Helsinki Sleep Clinic, Vitalmed Research Centre Helsinki and Medicum, Faculty of Medicine, University of Helsinki, Finland; e Infectious Disease Control and Vaccination Unit, Finnish Institute for Health and Welfare, Helsinki, Finland; f Reseach Program for Clinical and Molecular Metabolism, Faculty of Medicine, University of Helsinki; g Department of Bacteriology and Immunology and Translational Immunology Research Program, Faculty of Medicine, University of Helsinki, Helsinki, Finland; h Turku University Hospital, Clinical Microbiology, Kiinamyllynkatu 10, 20520, Turku, Finland

**Keywords:** Autoimmunity, OX_1_, OX_2_, Influenza A virus nucleoprotein, Narcolepsy, Pandemrix vaccination

## Abstract

Narcolepsy type 1, likely an immune-mediated disease, is characterized by excessive daytime sleepiness and cataplexy. The disease is strongly associated with human leukocyte antigen (HLA) DQB1∗06:02. A significant increase in the incidence of childhood and adolescent narcolepsy was observed after a vaccination campaign with AS03-adjuvanted Pandemrix influenza vaccine in Nordic and several other countries in 2010 and 2011. Previously, it has been suggested that a surface-exposed region of influenza A nucleoprotein, a structural component of the Pandemrix vaccine, shares amino acid residues with the first extracellular domain of the human OX_2_ orexin/hypocretin receptor eliciting the development of autoantibodies. Here, we analyzed, whether H1N1pdm09 infection or Pandemrix vaccination contributed to the development of autoantibodies to the orexin precursor protein or the OX_1_ or OX_2_ receptors. The analysis was based on the presence or absence of autoantibody responses against analyzed proteins. Entire OX_1_ and OX_2_ receptors or just their extracellular N-termini were transiently expressed in HuH7 cells to determine specific antibody responses in human sera. Based on our immunofluorescence analysis, none of the 56 Pandemrix-vaccinated narcoleptic patients, 28 patients who suffered from a laboratory-confirmed H1N1pdm09 infection or 19 Pandemrix-vaccinated controls showed specific autoantibody responses to prepro-orexin, orexin receptors or the isolated extracellular N-termini of orexin receptors. We also did not find any evidence for cell-mediated immunity against the N-terminal epitopes of OX_2_. Our findings do not support the hypothesis that the surface-exposed region of the influenza nucleoprotein A would elicit the development of an immune response against orexin receptors.

## Highlights

• No evidence of humoral immunity against human OX_1_ or OX_2_ orexin receptors.
• No cross-reactive antibodies between influenza virus NP and orexin receptors.
• No evidence for cell-mediated immunity against the N-terminal epitopes of OX_2_.

## Authors’ contributions

KM and IJ designed the study, interpreted the results and wrote the manuscript. PJ conducted EIA experiments and contributed to the writing of the manuscript. JPK constructed the expression constructs for OX_1_ and OX_2_, interpreted the results and contributed to the writing of the manuscript. MP, HN, AV and OV analyzed the patient data and contributed to the collection and analysis of serum specimens and writing of the manuscript. TLF and SM conducted T cell stimulation experiments, interpreted the data and contributed to writing of the manuscript.

## 1 Introduction

Narcolepsy, divided in two different disease subcategories, is a chronic neurological disorder of hypersomnolence. The main symptoms of narcolepsy type 1 (NT1) are excessive daytime sleepiness, cataplexy and disturbed nocturnal sleep. Patients may also have rapid eye movement sleep-related symptoms such as hypnagogic hallucinations and sleep paralysis [1,2]. In general, NT1 starts during the adolescence or young adulthood, and disease onset before the age of 10 years has been rare [[3], [4], [5]]. NT1 is strongly associated with human leukocyte antigen (HLA) class II DQB1∗06:02 [6,7]. An immune-mediated neuronal destruction, resulting in a deficit in the endogenous orexin (also called hypocretin) production in the lateral hypothalamus, is considered to be the primary pathophysiological mechanism of NT1 [[8], [9], [10], [11]]. The onset of NT1 has been shown to be seasonal, and in some studies NT1 has been associated with upper respiratory tract infections, such as H1N1 influenza A virus infection [12] and/or streptococcal infections [13,14]. This suggests that these infections could initiate an immune response that leads to the loss of orexin-secreting neurons in hypothalamus and the development of clinical NT1. Etiologically unknown narcolepsy type 2 is characterized by normal central nervous system orexin levels and absence of cataplexy.

**Fig. 1:**
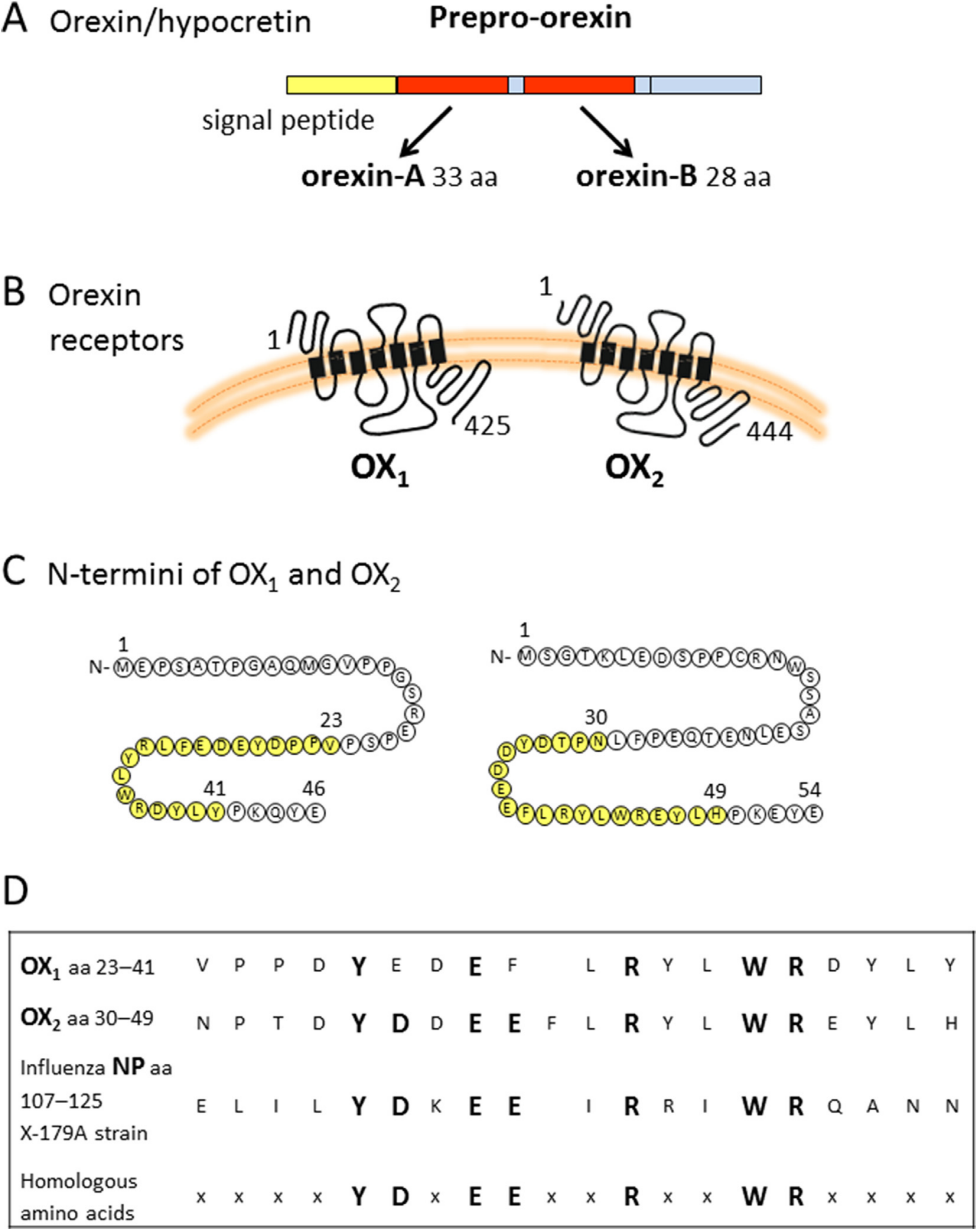
Peptide epitopes of interest for the current study. (A) Orexin-A and orexin-B are cleaved from their 131-amino acid-long precursor prepro-orexin that also contains an N-terminal signal peptide. Orexin-A contains two disulfide bridges. (B) Orexin receptors are transmembrane proteins located on cell surface. (C) OX_1_ has a 46-amino acid-long and OX_2_ a 54-amino acid-long N-terminus. The potential cross-reactive epitopes between OX_1_ and OX_2_ receptors and influenza A virus NP are shown in yellow. (D) An alignment of human OX_1_ and OX_2_ receptors and the influenza nucleoprotein (X-181 reassortant vaccine) used in Pandemrix vaccine show a consensus sequences between influenza NP and orexin receptors (identical amino acids are marked in large bold) (Adapted from Ahmed et al., 2015 [28]). The positions of the amino acids are as indicated in the figure.

Biologically active orexin peptides are produced by the cleavage of the 131-amino acid-long orexin precursor protein (prepro-orexin/preprohypocretin) into two smaller peptides: a 33-amino acid-long orexin-A/hypocretin 1 with two intrachain disulfide bonds and a 28-amino acid-long orexin-B/hypocretin 2 (Fig. 1). Prepro-orexin is produced in the lateral and posterior hypothalamus by a small number of specific neuronal cells. Orexin peptides act via two G-protein-coupled orexin receptors OX_1_ and OX_2_ [15]. OX_1_ and OX_2_ are 425- and 444-amino acid-long transmembrane receptors that have 46- and 54-amino acid-long N-terminal extracellular domains, respectively, followed by seven transmembrane regions, three intra- and three extracellular loops, and relatively short cytoplasmic tails (Fig. 1). The dual orexin/hypocretin nomenclature originates from the parallel discovery of the system by two different groups [15,16]. According to the current recommendations, which are followed in this paper, the peptides are to be called with the orexin nomenclature while the genes follow the hypocretin nomenclature (*HCRT* for the human peptide and *HCRTR1* and *HCRTR2* for the receptors).

Increased incidence of NT1 was observed in Finland, Sweden and France after vaccination of the general population with Pandemrix vaccine in 2009 [5,[17], [18], [19]]. Later, increased incidence of narcolepsy was also found in Norway, the United Kingdom, Ireland and Germany [[20], [21], [22], [23]]. It has been suggested that vaccination with AS03-adjuvanted Pandemrix vaccine, in the absence or possibly together with some other environmental factors, contributed to a 5 to 14-fold increased incidence of narcolepsy in HLA DQB1∗06:02-positive children and adolescents and the 2- to 7-fold increased incidence in adults [24].

The pH1N1-like vaccine strain X-179 ​A, used in the production of Pandemrix vaccine, was created as a reassortant virus using an H1N1-1918-like strain, A/Puerto Rico/8/1934 (PR8), as a backbone vaccine virus. Hemagglutinin (HA), neuraminidase (NA) and polymerase protein 1 (PB1) genes were derived from A/California/07/2009 virus and the remaining five viral genes from the PR8 virus [[25], [26], [27]]. Previously, it has been suggested that an epitope of the surface of influenza A PR8 virus nucleoprotein, used in Pandemrix, shows molecular mimicry with the extracellular N-terminus of the OX_2_ receptor [28]. It was hypothesized that this would trigger autoantibody formation and an immune-mediated neuronal destruction, resulting in a deficit in the endogenous orexin production in vaccinated HLA DQB1∗06:02-positive children and adolescents [28].

There is controversial information in the literature whether narcoleptic patients have autoantibodies against orexin receptors [[28], [29], [30], [31]]. Therefore, we analyzed whether Pandemrix-vaccinated narcoleptic children and adolescents and control individuals as well as patients who suffered a laboratory confirmed H1N1pdm09 virus infection have autoantibodies against human orexin or OX_1_ and OX_2_ receptors.

In this study, using an immunofluorescence microscopy assay (IFA), we analyzed human sera collected from 56 Pandemrix-vaccinated narcoleptic patients; 28 patients, who suffered from a laboratory-confirmed H1N1pdm09 infection; and 19 Pandemrix-vaccinated controls. Cell-mediated immunity against peptides derived from the N-terminus of the OX_2_ receptor was also analyzed in 6 NT1 patients and 5 Pandemrix-vaccinated controls. Our findings do not support the hypothesis that influenza H1N1pdm09 infection or vaccination with the Pandemrix vaccine induced cross-reactive autoantibodies or T cells against influenza A virus nucleoprotein (NP) and the N-terminus of OX_2_.

## 2 Materials and methods

### 2.1 Ethical permissions

The study protocol was approved by the Institutional Review Board of the National Institute for Health and Welfare (THL), Helsinki, Finland, and the Ethics committee of the Helsinki and Uusimaa Health District (permission code number 331/13/March 03, 2010) covering the participating hospitals. The collection of blood samples from narcoleptic children and Pandemrix-vaccinated controls have been previously described [32,33]. Informed written consent was given by the children and/or their guardians.

The collection of blood samples from individuals suffering from an upper respiratory tract infection was approved by the Ethics Committee of the Pirkanmaa Hospital District, Tampere, Finland (ETL-code numbers R09152 ​M and R10075 ​M). All patients gave their written informed consent for blood samples. The studies fully comply with the national and international regulations, privacy and data protection relevant to medical and patient-oriented studies.

### 2.2 Identification of narcoleptic patients

The present study comprises 56 narcoleptic patients aged 5–18 ​years ​at the onset who fell ill in 2009 or 2010 after receiving Pandemrix vaccine in November or December 2009. The description of the patients, the date of Pandemrix vaccination, time of the onset of narcolepsy and the time of serum sample collection have been previously described [17,[32], [33], [34]]. The diagnosis of narcolepsy with cataplexy was based on the 2005 version of International Classification of Sleep Disorders (ICSD-2) [1]. All our diagnoses have been later reclassified into type 1 narcolepsy (ICD-10 code G47.4) according to the current (ICSD-3) version of the classification [35]. A polysomnography and Multiple Sleep Latency Test (MSLT) and HLA DQB1 genotyping with a panel of sequence-specific oligonucleotide probes [36] were carried out for each patient. If the diagnosis of narcolepsy with cataplexy was not clear, also a lumbar puncture was done with analysis of cerebrospinal fluid orexin-levels according to the Stanford RIA protocol to verify the diagnosis. Other causes of excessive daytime sleepiness (EDS) (e.g. sleep apnea, delayed sleep phase syndrome or sleep deprivation) as well as other neurological disorders (encephalitis, encephalopathy, other neurological disorders) were excluded by polysomnography, actigraphy, thorough neurological examination, magnetic resonance imaging (MRI), electroencephalography (EEG), cerebrospinal fluid (CSF) examinations, blood tests, and other examinations when necessary. Based on the review of patient records, an experienced neurologist and a sleep specialist (Drs. Markku Partinen and Turkka Kirjavainen), independently of each other, validated the time of onset and diagnosis of narcolepsy. If the opinion of the two specialists differed significantly, the onset time and diagnosis was verified by a panel of three neurologists/sleep specialists (alternating Drs. C. Hublin, O. Saarenpää-Heikkilä, P. Olsén and S.-L. Himanen).

### 2.3 Blood samples from narcoleptic patients and from healthy controls

Blood specimens from all 56 narcoleptic patients and 19 control individuals were collected during 2011. Paired serum specimens from 28 adults suffering from a clinical, laboratory-confirmed H1N1pdm09 virus infection were collected during the epidemic seasons 2009–2010 and 2010–2011 in Tampere, Finland. H1N1pdm09 infection was confirmed by identifying viral RNA in nasopharyngeal stick samples collected during the acute phase of the disease using a diagnostic polymerase chain reaction test with the NS gene as the target [37]. Five of the 28 patients had previously been vaccinated with Pandemrix vaccine.

Paired serum specimens were collected at the acute and convalescent phases (14–21 days later) of the disease. All patients showed a diagnostic antibody rise in the H1N1pdm09 virus-specific hemagglutination inhibition assay (HI; ≥ 4 ​× ​rise) or H1N1pdm09 NS1 protein-specific Western blot assay (≥2 ​× ​rise) [33].

### 2.4 Patient and healthy control donor material in the T cell study

The T cell study included 6 pediatric Pandemrix-associated NT1 patients and 5 healthy Pandemrix-vaccinated control children or adolescents. NT1 patients were diagnosed in neurological outpatient clinics at Finnish hospitals and health care centers by neurologists with expertise in sleep medicine. All patients were diagnosed with NT1 based on criteria defined in the 3rd edition of the International classification of sleep disorders [35].

### 2.5 Cells

Human hepatocellular carcinoma HuH7 [38] cells were maintained in continuous culture in minimum essential medium (MEM) (Thermo Fisher Scientific Inc., Waltham, MS, USA) supplemented with 0.6 ​μg/ml penicillin, 60 ​μg/ml streptomycin and 10% fetal calf serum (Sigma-Aldrich Co. LLC., St. Louis, MO, USA). *Spodoptera frugiperda* 9 (*Sf*9) cells were used for baculovirus expression and maintained in TNM-FH (Sigma-Aldrich) medium as described previously [39]. To collect human peripheral blood mononuclear cells (PBMC), whole blood was drawn and heparinized. PBMCs cells were isolated by Ficoll gradient centrifugation (GE Healthcare, Uppsala, Sweden) and stored in liquid nitrogen until further analysis.

### 2.6 T cell peptides

Five peptides covering the amino acid sequence of the human OX_2_ from amino acid positions 27 to 53 (Fig. 1) were produced, including the core peptide DYDDEEFLRYLWREY (15-mer peptides, 12 amino acids overlap; New England Peptides, Gardner, MA, USA). The peptides were dissolved in endotoxin-free water/DMSO (50%; both from Sigma-Aldrich Co.) and stored as 25 ​mM stock at −70 ​°C, until the day of use.

### 2.7 PBMC stimulation experiments

Frozen PBMCs were thawed and allowed to recover in RPMI 1640 culture medium (Thermo Fisher Scientific Inc.) containing 2 ​mM l-glutamine, 25 ​mM HEPES, 25 ​μg/ml gentamycin (Sigma-Aldrich) and 5% of heat-inactivated serum from AB-negative individuals (Innovative Research, Novi, MI, USA) at 37 ​°C in a CO_2_ incubator for 1 ​h. The cells were stimulated with the 15-mer peptides based on the N-terminus of the OX_2_ receptor (10 ​μM each; resulting DMSO concentration 0.1%), tetanus toxoid (20 ​μg/ml), or PBS (negative control) for 6 days.

### 2.8 Antibodies and immunofluorescence (IFA)

For immunofluorescence microscopy, studied human sera, rabbit polyclonal anti-OX_2_ immunoglobulins (AB3094; EMD Millipore Corp., Temecula, CA, USA), primary mouse monoclonal anti-His immunoglobulins (sc-53073; Santa Cruz Biotechnology, Santa Cruz, CA, USA) and rabbit anti-influenza A/PR/8/34 virus NP were diluted 1:25 and 1:100 (patient sera), 1:200 (anti-OX_2_), 1:200 (anti-His) or 1:250 (anti-NP) into 0.5% bovine serum albumin (BSA) in PBS and incubated at 4 ​°C overnight. Influenza A NP was produced in *E. coli*; the method for antibody production has been described previously [40]. Secondary Texas Red-labeled goat anti-human (Vector, Vector Laboratories, Inc., Burlingame, CA, USA), rhodamine-labeled goat anti-rabbit (#111-026-047, Jackson ImmunoResearch Laboratories, Inc., West Grove, PA, USA) and fluorescein-labeled goat anti-mouse (#116-095-003, Jackson ImmunoResearch Laboratories) immunoglobulins were used according to the manufacturers’ instructions. After washing, the glass coverslips were mounted on Mowiol (Sigma-Aldrich Co.) and images were taken with Leica TCS NT confocal microscope.

### 2.9 Plasmids and DNA manipulations

The OX_1_-GFP baculovirus expression vector has been described previously [41]. The OX_2_-GFP baculovirus was constructed in the same way by Drs. Johnny Näsman (Åbo Akademi University, Turku, Finland) and Jaana Putula (University of Helsinki) from the OX_2_-GFP sequence as described in Ref. [42].

For the creation of OX_1_-and OX_2_-GFP-V5-His as well as OX_1_-and OX_2_-GFP expression constructs, cDNAs coding for the human OX_1_ and OX_2_ receptors [42] were first digested with *Bsr*GI, blunted by Klenow fragment fill-in and then digested with *Hind*III. The fragments were ligated into pcDNA3.1/V5-His TOPO (Invitrogen) digested with *Hind*III–*Eco*RV.

Synthetic genes coding for the N-termini of the human OX_1_ (amino acids 1–46; cDNA of *HCRTR1*: NM_001525.2) and OX_2_ receptors (amino acids 1–54; cDNA of *HCR*TR2: NM_001526.4) and the full-length prepro-orexin (amino acids 1–131; cDNA of *HCRT*: NM_001524.1) were ordered from GenArt (Thermo Fisher Scientific Inc.). The 5′-ends were modified by adding a *Bgl*II/*Bam*HI restriction site and a Kozak consensus sequence, ACC, prior to the translation start sites (AGA TCT GGA TCC ACC ATG, initiation codon underlined). To the 3′-ends of the cDNAs, a second *Bgl*II/*Bam*HI site, without a stop codon, was added, enabling the subcloning of the genes into the *Bam*HI site of the eukaryotic expression vector pcDNA3.1 (−)A-*myc*-His (Invitrogen).

Influenza A/WSN/33 NP (accession number: CY034135.1) gene was modified by PCR to create 5′ and 3′ *Bam*HI sites and an N-terminal Kozak consensus sequence for its further cloning into the eukaryotic expression vector pcDNA3.1 (+) (Thermo Fisher Scientific Inc.).

All DNA manipulations were performed according to standard protocols, and the newly created gene constructs were fully sequenced.

### 2.10 Production of OX_1_-GFP- and OX_2_-GFP-expressing baculoviruses

Three days after infecting *Sf*9 cells with OX_1_-GFP- and OX_2_-GFP-expressing baculoviruses, cell medium was collected and centrifuged in 2 ​ml tubes at 12 ​000 ​rpm for 30 ​min. Supernatants were collected and baculovirus stocks were diluted in 1:10 volume of EMEM supplemented as mentioned above. For further transduction experiments, baculoviruses were stored at 4 ​°C.

### 2.11 Transduction and transfection of HuH7 cells

HuH7 cells were first transduced for 1 ​h with OX_1_-GFP- and OX_2_-GFP-expressing baculoviruses in EMEM supplemented with 2% fetal calf serum, followed by removal of the viruses and addition of fresh growth medium supplemented as above. Alternatively, HuH7 cells were grown on glass coverslips for 24 ​h and then transfected with expression plasmids (expressing OX_1_-GFP-V5-His, OX_2_-GFP-V5-His, OX_1_ (1–46)-*myc*-His, OX_2_ (1–54)-*myc*-His, prepro-HCRT-*myc*-His or influenza A/WSN/33 NP) using *Trans*IT®–LT1 transfection reagent (Mirus Bio Corp., Madison, WI, USA) according to the manufacturer’s instructions. At 24 ​h post transduction/transfection, the cells were fixed with 3% paraformaldehyde in PBS for 30 ​min, permeabilized with 0.1% Triton X-100 in PBS for 15 ​min and blocked with 0.5% BSA in PBS at RT for 30 ​min.

### 2.12 Enzyme immunoassay (EIA), antigens and statistical analysis

Production of GST and GST-NP (A/PR/8/34) has been described elsewhere [40]. H1N1 antigen suspension of Pandemrix (GlaxoSmithKline, Dresden, Germany) vaccine was concentrated with Vivaspin 10 ​K PES membrane filters (Sartorius, Darmstadt, Germany).

Influenza A nucleoprotein or H1N1 vaccine virus-specific antibody EIA was done as described before [43]. Briefly, 96-well microtiter plates (Nunc Maxisorp, Thermo Fisher Scientific Inc.) were coated with 0.5 ​μg/ml of GST, 1.5 ​μg/ml of GST-NP and 1.0 ​μg/ml of H1N1 antigen suspension of Pandemrix vaccine in PBS at 4 ​°C overnight. Plates were stored at 4 ​°C and washed once with washing buffer (0.05% Tween-20 in PBS) before starting the assay. Serum samples were diluted 1:500 in sample buffer (5% swine serum (Biological Industries, Beit Haemek, Israel), 0.1% Tween-20 in PBS) and incubated at 37 ​°C for 2 ​h. After washing, secondary horseradish peroxidase (HRP) labeled anti-human IgG (Dako A/S, Glostrup, Denmark) antibody was diluted 1:8000 in sample buffer and incubated for 1 ​h ​at 37 ​°C. TMB One (Kementec Solutions A/S, Taastrup, Denmark) was used as substrate and the reaction was stopped with 0.1 ​M ​H2SO4 after 20 ​min. Absorbance was measured at 450 ​nm with Victor Nivo plate reader (PerkinElmer Inc., Waltham, MS, USA). Results are expressed as EIA units using seronegative and highly positive serum samples as standards [43].

Statistical differences in IgG antibody levels between patient and control groups were analyzed with one-way ANOVA. Calculations and graphs were done using GraphPad Prism 8 software and p-values of <0.05 were considered statistically significant.

### 2.13 Cytokine fluorescent multiplex bead-based immunoassay (FMIA)

Interferon-gamma (IFN-γ) release to the cell culture medium was measured using the human Milliplex MAP Kit (HCYTMAG-60 ​K, Millipore, Billerica, MA, USA), according to manufacturer’s instructions. Quantification was performed with a Bio-Plex 200® System and Bio-Plex Manager software version 5.0 (BIO-RAD Laboratories, Hercules, CA, USA). Each sample was compared to a negative control.

## 3 Results

### 3.1 No serological evidence of autoantibodies to OX_1_ or OX_2_ receptors in Pandemrix-vaccinated narcoleptic children or in influenza A virus-infected patients

**Fig. 2:**
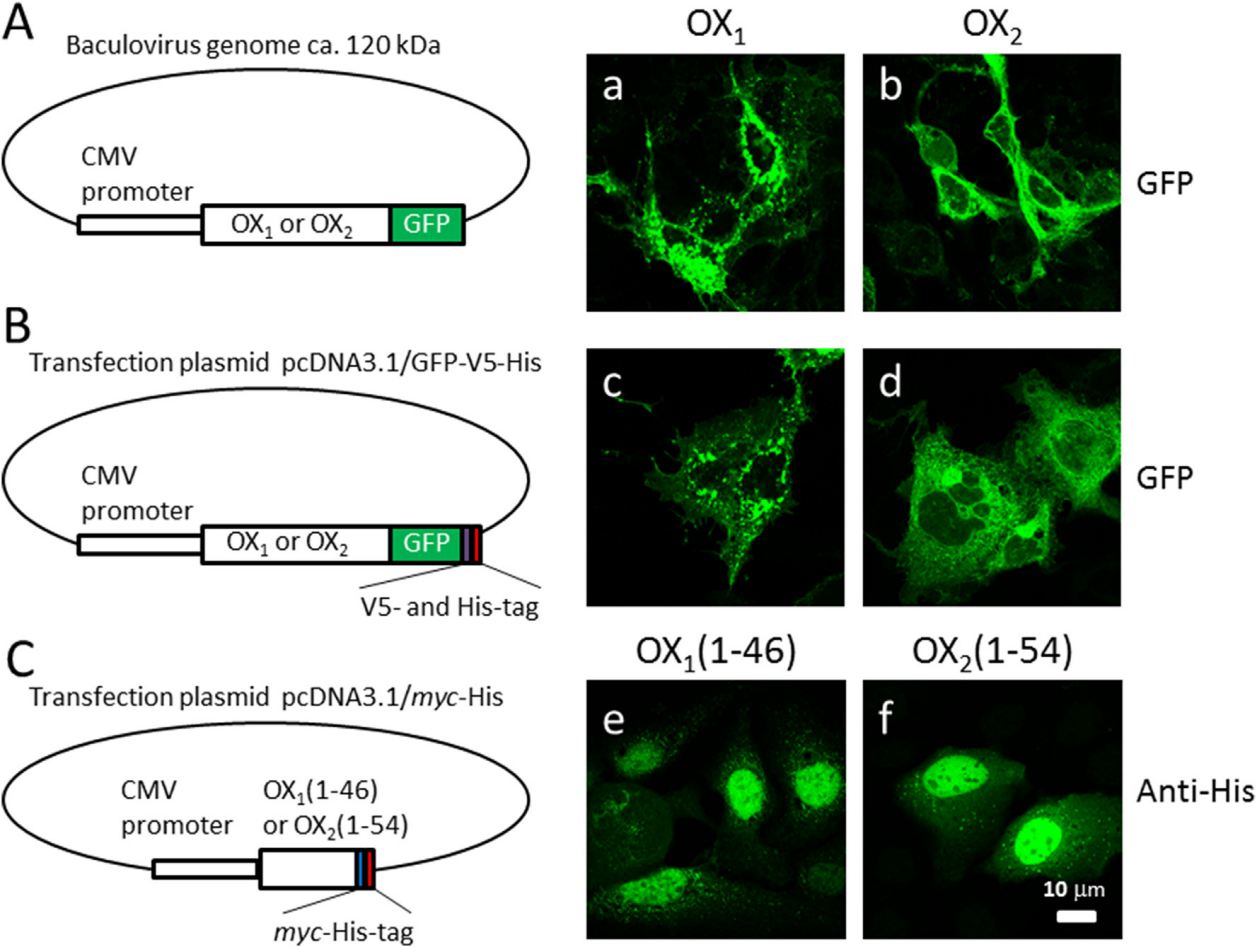
Three different vector constructs used to express OX_1_ and OX_2_ receptor fusion and deletion proteins. (A) OX_1_-GFP and OX_2_-GFP, under a CMV promoter, were expressed in HuH7 cells using a baculovirus-mediated gene delivery method. Panels a and b show the cellular expression pattern of OX_1_-GFP and OX_2_-GFP, respectively, in HuH7 cells. (B) OX_1_-GFP-V5-His and OX_2_-GFP-V5-His, under the CMV promoter in the pcDNA3.1 expression vector, were transiently transfected into HuH7 cells. Panels c and d show the expression pattern of OX_1_-GFP-V5-His and OX_2_-GFP-V5-His in HuH7 cells, respectively. (C) The N-termini of the OX_1_ and OX_2_ receptors, fused to the *myc* and His tags, were transiently transfected into HuH7 cells. Panels e and f show the intracellular location and expression pattern of the N-termini of OX_1_ and OX_2_ receptors, respectively, in HuH7 cells. The calibration bar applies to all images.

**Fig. 3:**
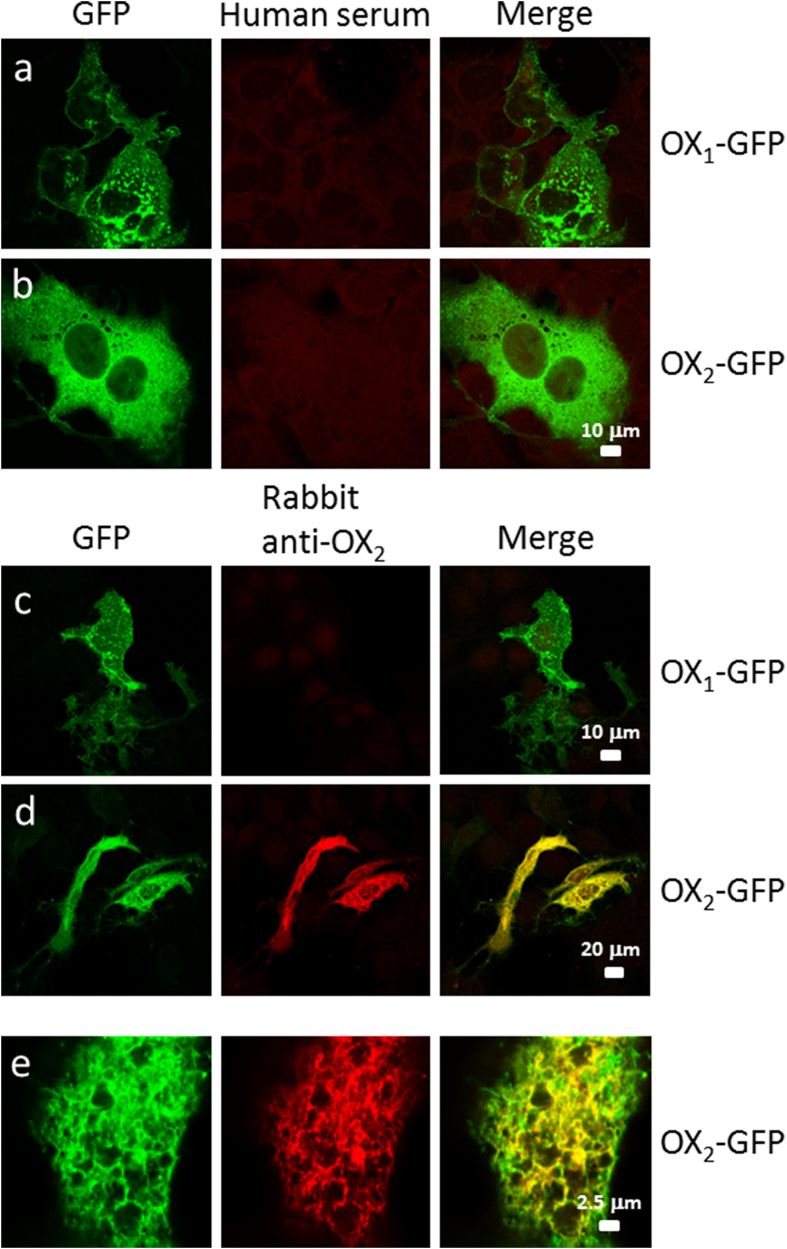
Human sera do not recognize OX_1_ or OX_2_ receptors while rabbit polyclonal anti-OX_2_ immunoglobulins recognize OX_2_ in baculovirus-transduced HuH7 cells. HuH7 cells, grown on glass coverslips, were first transiently transduced with baculoviruses coding for OX_1_-GFP or OX_2_-GFP under CMV promoter. After 24 ​h, the cells were fixed and stained with 1:25 dilutions of human narcoleptic patient sera, followed by staining with Texas Red-labeled anti-human immunoglobulins (rows a and b) or with 1:200 diluted rabbit anti-OX_2_ immunoglobulins, followed by staining with Texas Red-X-labeled anti-rabbit immunoglobulins (rows c, d and e) as indicated in the figure. In the merged images, yellow indicates colocalization of GFP and rabbit anti-OX_2_ antibodies. The same calibration bar applies to all images in a and b, and for c–e, for the entire row. Panel e represents larger magnification of stained cells.

**Fig. 4:**
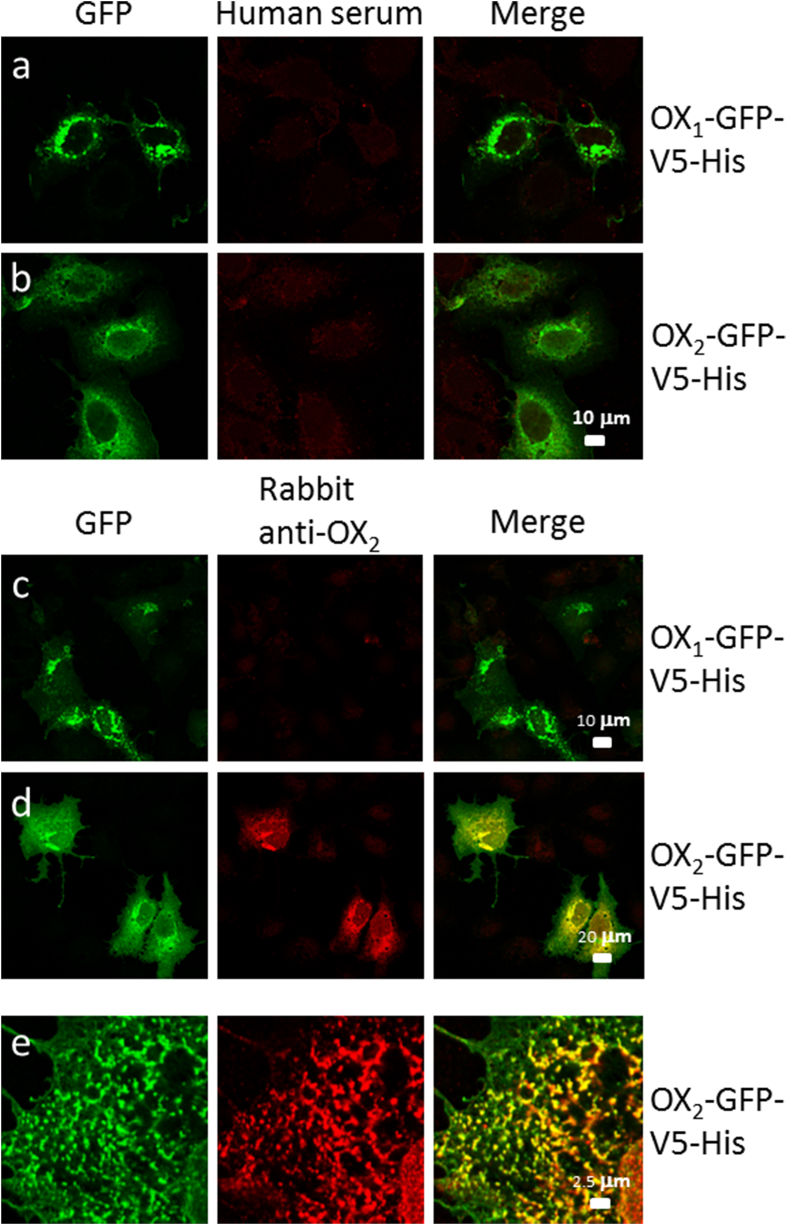
Analysis of IgG antibodies against OX_2_ in transiently transfected HuH7 cells. HuH7 cells, grown on glass coverslips, were first transiently transfected with OX_1_-GFP-V5-His or OX_2_-GFP-V5-His expression constructs. After 24 ​h, the cells were fixed and stained with 1:25 diluted human narcoleptic patient sera, followed by staining with Texas Red-labeled anti-human immunoglobulins (rows a and b) or with 1:200 diluted rabbit anti-OX_2_ immunoglobulins, followed by staining with Texas Red-X-labeled anti-rabbit immunoglobulins (rows c, d and e) as indicated in the figure. In merged images, yellow indicates colocalization of GFP and rabbit anti-OX_2_ antibodies. The same calibration bar applies to all images in a and b, and for c–e, for the entire row. Panel e represents larger magnification of stained cells.

We either transduced HuH7 cells with baculoviruses, which were modified to produce OX_1_-GFP or OX_2_-GFP in eukaryotic cells (Fig. 2A), or transfected the cells with OX_1_-GFP-V5-His or OX_2_-GFP-V5-His gene expression constructs (Fig. 2B). OX_1_ and OX_2_ receptors showed similar membranous expression pattern in Huh7 cells where the expression was induced by baculovirus transduction or gene transfection. Both OX_1_ and OX_2_ were expressed in cellular membranes and ER-like structures, however, OX_1_ also seemed to form larger granular structures (Fig. 2a-d). OX_1_ and OX_2_ are transmembrane proteins (Fig. 1B) and autoantibodies against these proteins can be screened by staining OX_1_-GFP- and OX_2_-GFP-producing cells with human sera followed by secondary anti-human IgG rhodamine conjugate and analyzing the staining patterns with confocal laser microscopy. Serum specimens were analyzed in 1:25 and 1:100 dilutions to ensure a sensitive detection of also low antibody levels. None of the analyzed patient/vaccinee sera showed autoantibodies to OX_1_ or OX_2_ receptors (Fig. 3, Fig. 4a,b). However, polyclonal rabbit anti-OX_2_-specific immunoglobulins readily detected transiently expressed OX_2_ gene products in HuH7 cells (Fig. 3, Fig. 4e) as evidenced with a clear colocalization of GFP (green) and rhodamine-labeled (red) rabbit anti-OX_2_ IgG antibodies in confocal microscopy (Fig. 3, Fig. 4).

### 3.2 No serological evidence of autoantibodies to the isolated N-termini of OX_1_ or OX_2_ receptors in Pandemrix-vaccinated narcoleptic children and control individuals or in influenza A virus-infected patients

**Fig. 5:**
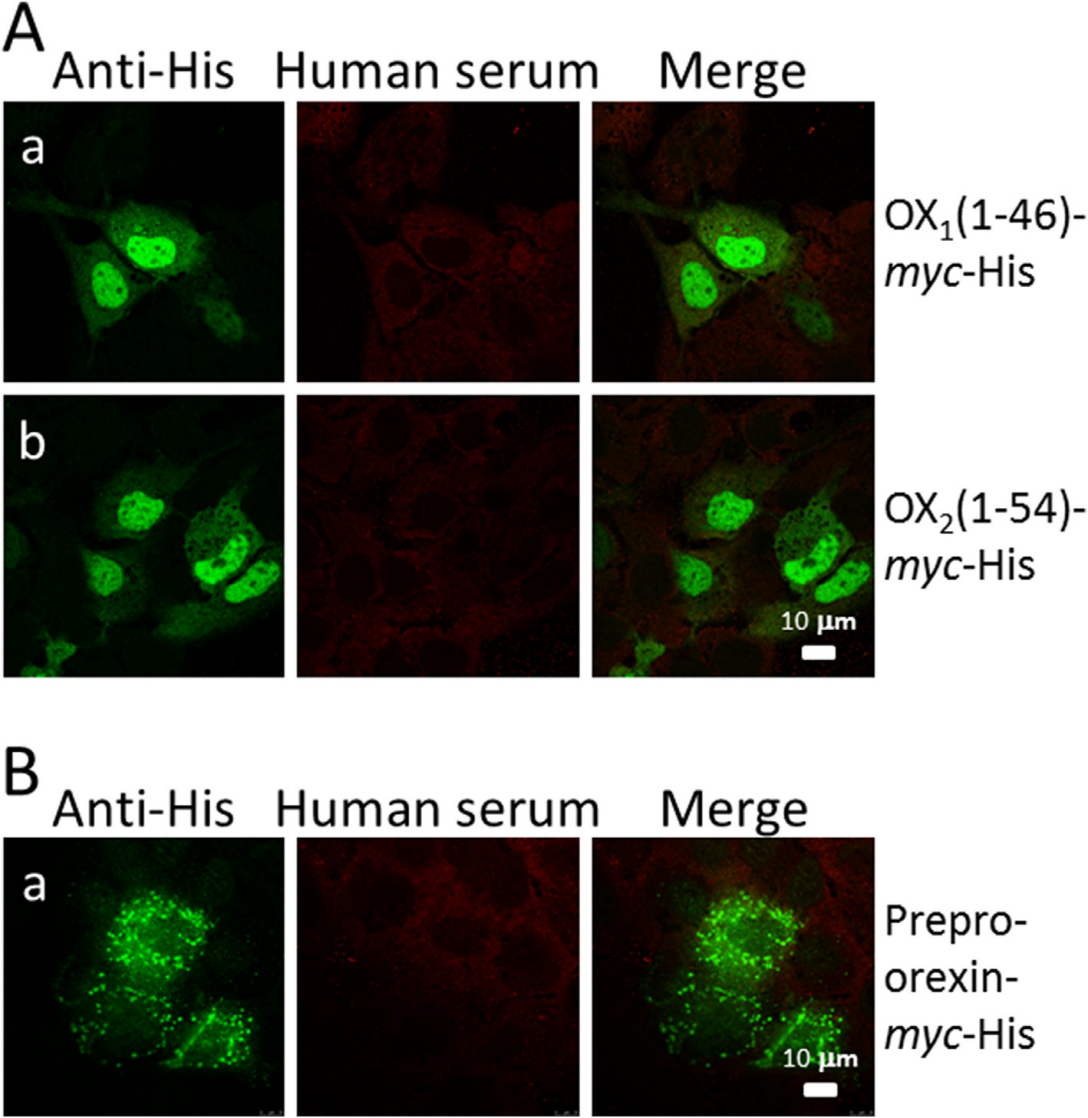
Human sera do not recognize the N-termini of OX_1_ or OX_2_ receptors or prepro-orexin in transiently transfected HuH7 cells. (A) HuH7 cells, grown on glass coverslips, were first transiently transfected with vectors expressing either N-terminal OX_1_ (1–46)-*myc*-His or OX_2_ (1–54)-*myc*-His. After 24 ​h, the cells were fixed and stained with 1:25 diluted human narcoleptic patient sera and with 1:200 diluted mouse anti-His immunoglobulins followed by staining with Texas Red-labeled anti-human and FITC-labeled anti-mouse immunoglobulins as indicated in the figure (rows a and b). (B) HuH7 cells were first transiently transfected with prepro-orexin-*myc*-His expression vector and then fixed and stained as above (row a). Colocalization is visualized as yellow color (merged image). The same calibration bar applies to all images.

Next, we analyzed the possible presence of autoantibodies to OX_2_ receptors from the same sera as above using a modified experimental protocol. We transiently transfected HuH7 cells with expression vectors encoding the N-terminal parts of human OX_1_ (amino acids 1–46) or OX_2_ (amino acids 1–54) receptors fused to C-terminal *myc*-His-tags. This enabled us to double stain the cells with patient sera and anti-His immunoglobulins, and to analyze the staining patterns with confocal laser microscopy. We analyzed all 56 sera from narcoleptic children, paired serum specimens from 28 adults and 19 sera from Pandemrix vaccinated controls as above. Dilutions of 1:25 and 1:100 of the sera were used. None of these sera showed autoantibodies to OX_1_ or OX_2_ receptor N-terminus (Fig. 5A).

### 3.3 No detectable autoantibodies to the full-length prepro-orexin in Pandemrix-vaccinated narcoleptic children, control individuals or influenza virus-infected patients

We also analyzed possible presence of autoantibodies to the orexin precursor, prepro-orexin, from the same sera as above. We transiently transfected HuH7 cells with cDNA, encoding the full-length 131-aa-long prepro-orexin, subcloned into *myc*-His-tagged expression vector in which the tags were expressed in the C-terminal end of the molecule. This enabled us to use double staining with patient sera and anti-His immunoglobulins and analyze staining patterns with confocal laser microscopy. We analyzed 46 serum specimens from narcoleptic children, paired serum specimens from 28 adults and 19 sera from Pandemrix vaccinated controls as above. The dilution of human sera was 1:25. None of the analyzed sera showed autoantibodies to the orexin precursor molecule (Fig. 5B).

### 3.4 All analyzed human sera had antibodies against influenza A virus NP detected by EIA and IFA

We also wanted to analyze, whether narcoleptic patients and controls had antibodies to some influenza A virus proteins, such as NP, as an indication of previous suffered influenza A virus infection and/or efficient anti-NP response induced by the Pandemrix vaccine. NP was chosen as the target viral protein, since it is likely that all individuals develop antibodies against this protein and it is genetically very stable. We used both IFA and EIA to determine the IgG antibody levels against NP. In addition, we used EIA to detect IgG antibody levels against H1N1 antigen suspension of Pandemrix vaccine.

**Fig. 6:**
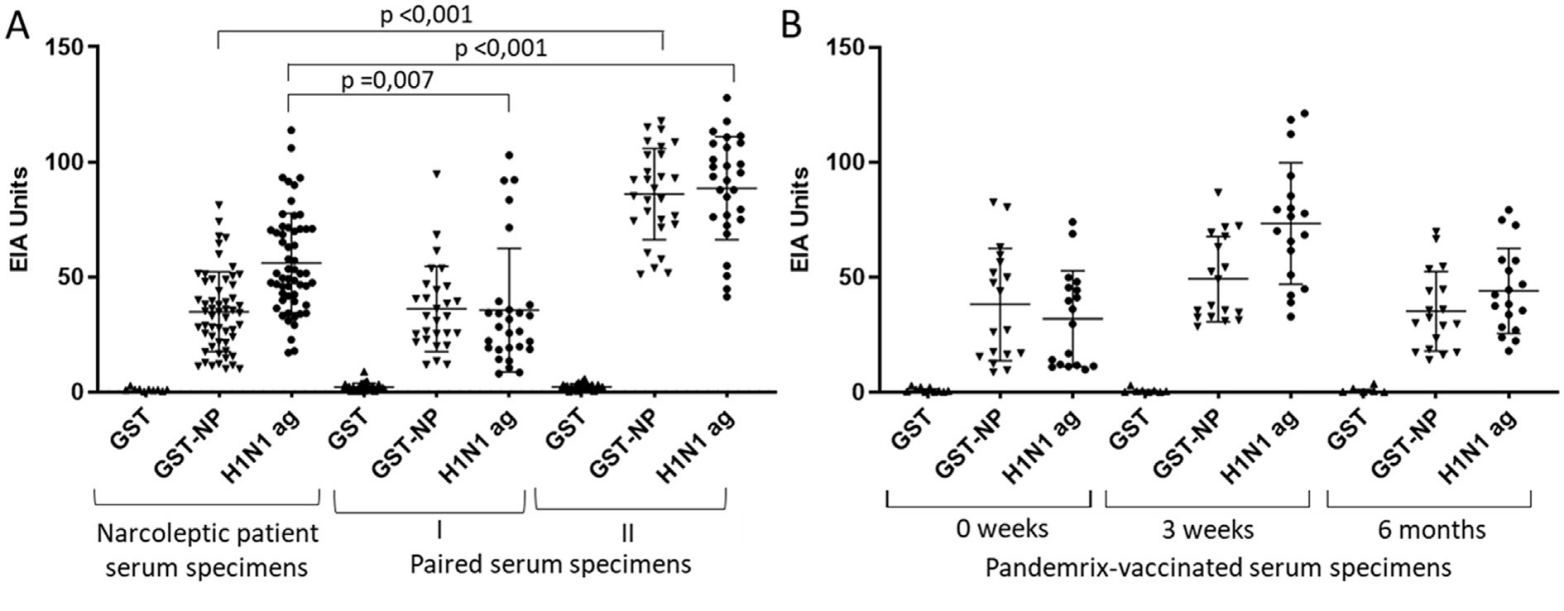
IgG antibody levels against GST protein (negative control), IAV NP with GST protein and H1N1pdm09 antigen suspension of Pandemrix vaccine determined by EIA. A) IgG levels in Pandemrix-vaccinated narcoleptic patient serum specimens (n ​= ​56) and in paired serum specimens collected in acute (I; n ​= ​28) and convalescent (II; n ​= ​28) phase of PCR-confirmed H1N1pdm09 virus infection. B) IgG levels in Pandemrix-vaccinated serum specimens collected before (n ​= ​18), 3 weeks after (n ​= ​18) and 6 months after (n ​= ​18) vaccination. Significance (p) values are shown between different groups although the groups are not directly comparable for anti-influenza antibody levels.

**Fig. 7:**
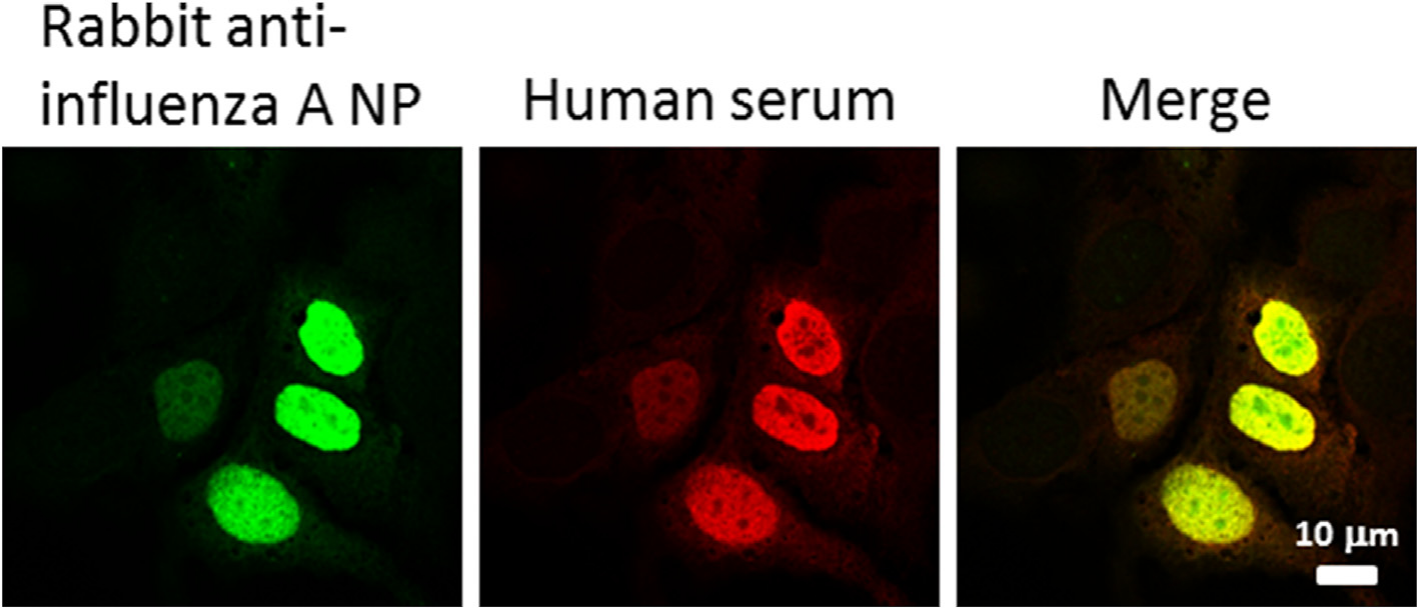
Antibodies in sera from influenza virus-infected patients and narcolepsy patients recognize influenza A virus NP in NP-expressing HuH7 cells. HuH7 cells, grown on glass coverslips, were first transiently transfected with a vector expressing influenza A WSN/33 NP gene. After 24 ​h, the cells were fixed and stained with 1:200 diluted rabbit anti-NP immunoglobulins and 1:100 diluted human sera, followed by staining with FITC-labeled anti-rabbit and Texas Red-labeled anti-human immunoglobulins. Merge images indicate double staining and colocalization of rabbit anti-NP and human anti-NP antibodies. The same calibration bar applies to all images.

In EIA, all analyzed serum specimens had anti-NP and anti-H1N1 antibodies but no activity against GST protein (Fig. 6). Both anti-NP and anti-H1N1 IgG-antibody levels increased after H1N1pdm09 virus infection and after receiving Pandemrix vaccine as expected. For IFA, we transiently transfected HuH7 cells with the influenza A/WSN/33 *NP* expression vector and analyzed 20 serum specimens from narcoleptic patients and controls (Fig. 7). All 20 analyzed sera were strongly anti-NP antibody positive.

The result allowed us to conclude that influenza A NP and H1N1 antigen suspensions were immunogenic, and the studied sera contained specific antibodies against influenza virus proteins (Fig. 6, Fig. 7). This confirmatory analysis indicated that immunofluorescence microscopic analysis is a reliable method to study whether human sera have antibodies against given protein structures.

### 3.5 T cell study

**Fig. 8:**
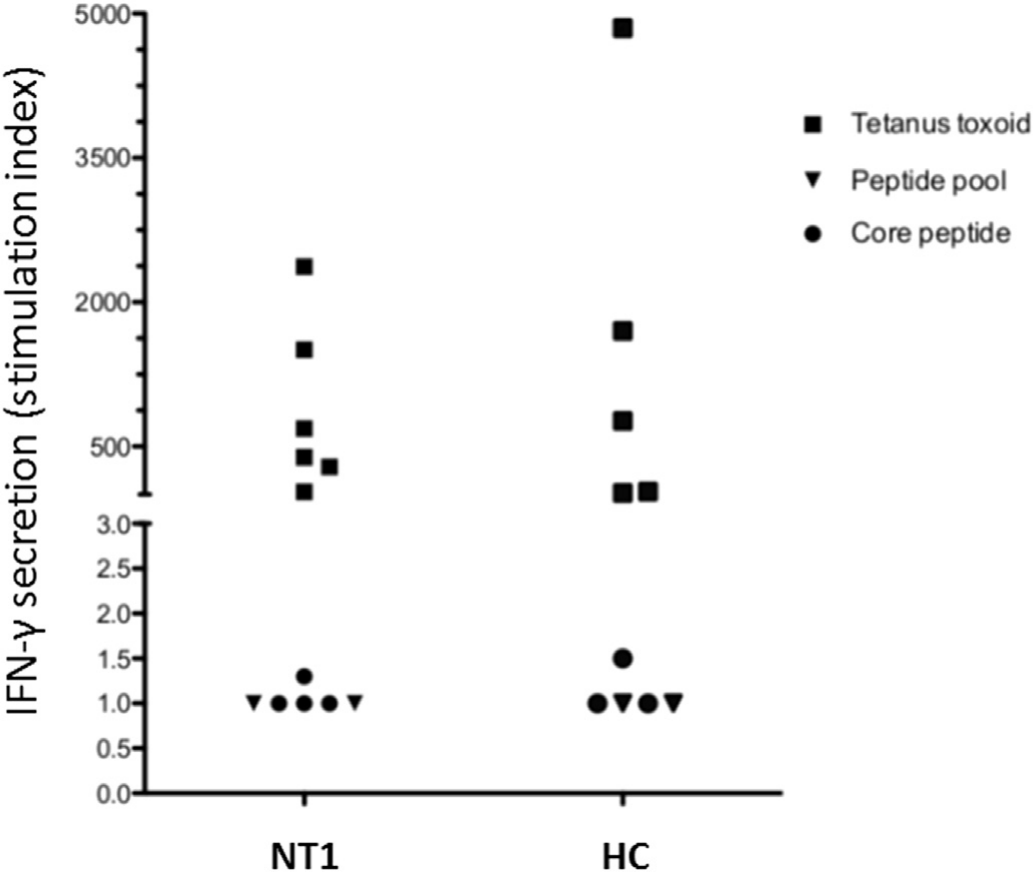
No cell-mediated immunity against isolated OX_2_ receptor N-terminal epitopes. Six Pandemrix-associated NT1 patient and five Pandemrix-vaccinated healthy control (HC) PBMC samples were stimulated for 6 days with tetanus toxoid (positive control), the OX_2_ receptor core peptide DYDDEEFLRYLWREY (OX_2_ 33–47), or a peptide pool of five 15-mer peptides, covering OX_2_ (27–53) epitopes with 12 amino acid overlaps (and including the core peptide DYDDEEFLRYLWREY). Secretion of IFN-γ to PBMC culture medium was measured by FMIA. Results are expressed as the ratio between a stimulated sample and the corresponding negative control (stimulation indices). Stimulation of PBMC samples with the OX_2_ N-terminus core peptide DYDDEEFLRYLWREY or with the peptide pool failed to induce IFN-γ production.

Since HLA class II antigen DQB1∗06:02 is strongly associated with narcolepsy, we analyzed the potential existence of cell-mediated immunity against the extracellular N-terminal epitope spanning from aa 27 to 53 of OX_2_ (Fig. 1). PBMC samples from six narcoleptic patients and five controls were stimulated with the core peptide DYDDEEFLRYLWREY (aa 33–47) showing partial homology to influenza A nucleoprotein [28], or with an OX_2_ peptide pool including DYDDEEFLRYLWREY and PBMC-produced IFN-γ was analyzed by FMIA. None of the narcoleptic patients or control showed stimulatory responses to OX_2_-specific peptides while all samples showed strong cell-mediated responses, i.e. high stimulatory indices in IFN-γ production in response to stimulation with tetanus toxoid (Fig. 8).

## 4 Discussion

All our narcolepsy patients related to Pandemrix vaccination were classified as narcolepsy type 1 [24]. Genotyping of the patients revealed them to be HLA DQB1∗06:02 positive, which is the strongest genetic factor predisposing to NT1 [6,7]. NT1 is characterized by the destruction of hypothalamic orexin-producing neurons, which leads to orexin deficiency in the central nervous system and development of clinical narcolepsy. At present, the environmental factors contributing to the pathophysiology of the disease have remained elusive. Cell-mediated autoimmunity and/or cytotoxicity caused by cytokines and inflammatory cells have been speculated to lead to the death of orexin-producing neurons in the hypothalamus [2,44]. Also autoantibodies against many types of tissues and molecules, including anti-Tribbles homolog 2 antibodies [45,46], anti-ganglioside antibodies [47], anti-neurexin-1 antibodies [47,48], and antibodies against various hypothalamic and neuronal structures [49] as well as antibodies against OX_2_ receptor have been identified [28]. The role of humoral autoimmunity in the onset of narcolepsy is unclear.

An increased risk to develop NT1 after pH1N1 vaccination has been linked to European Pandemrix vaccine but not to the Arepanrix vaccine used in Canada [17,24,34,50]. Vaarala and coworkers suggested that pandemic influenza vaccine-associated risk could be due to some antigenic differences in the H1N1 antigen used in the two vaccines, Pandemrix and Arepanrix [32]. The authors showed that the amounts of viral NP protein and its polymeric form were higher in the Pandemrix than in the Arepanrix vaccine. They also showed increased antibody responses against NP in patients positive in the DQB1∗06:02 allele [32].

It has been suggested that molecular mimicry and cross-reactivity between microbial and host proteins may contribute to the development of autoimmune diseases such as narcolepsy. Several groups have tried to detect autoantibodies against orexin receptors. In a limited number of sera, collected from children and adolescents who developed narcolepsy after Pandemrix vaccination, Ahmed and co-workers found antibodies against influenza A virus NP epitope, which cross-reacted with an epitope within the extracellular N-terminus of the human OX_2_ receptor [28]. However, other groups have been unable to confirm these results fully or at all. Using mass spectrometric analyses, Luo and co-workers failed to detect OX_2_-specific cross-reactive autoantibodies in any of the serum specimens of forty post-Pandemrix narcolepsy cases, suggesting that OX_2_-specific autoantibodies are not a characteristic feature of Pandemrix-associated narcolepsy [29]. Using a cell-based immunofluorescence method Ciannoccaro and co-workers found only low levels of IgG antibodies against OX_2_ in 3 out of 61 patients with narcolepsy, suggesting that autoantibodies against OX_2_ are uncommon in NT1 patients whose disease is not associated with the Pandemrix vaccination [30].

In the present study, our purpose was to further evaluate the molecular mimicry hypothesis of autoantibodies to OX_2_ in sera collected from Pandemrix-vaccinated narcoleptic children and adolescents. In this analysis immunofluorescence microscopy, with a laser confocal microscopic device, was selected for its sensitivity and specificity. We transiently expressed full-length human OX_1_ and OX_2_ receptors or just the N-terminal extracellular OX_1_ (amino acids 1–46) and OX_2_ (amino acids 1–54) receptor domains. HuH7 cells were also transiently transfected with a prepro-orexin expression construct. The use of chimeric GFP gene constructs with the full-length receptors enabled us to avoid double staining and a plausible cross-reactivity due to secondary antibodies. In double staining experiments we also failed to see any colocalization of patient serum immunoglobulins with His-tagged target molecules.

Both transduced and transfected full-length OX_1_-GFP and OX_2_-GFP expression constructs presented very similar staining patterns, indicating proper protein folding and likely correct expression of the receptors. However, short N-terminal OX_1_-*myc*-His and OX_2_-*myc*-His peptides were localized mainly into the cell nucleus. Since these molecules are small they are likely passively translocated into the nucleus.

Analysis of serum specimens from Pandemrix-vaccinated narcoleptic patients and controls as well as from patients, who suffered from a laboratory-confirmed H1N1pdm09 infection, failed to reveal any autoantibodies against OX_2_ using IFA. We were, thus, not able to confirm the results of Ahmed and co-workers, who found antibodies against influenza NP epitope, which cross-reacted with the N-terminus of human OX_2_ [28]. Our results are thus in line with the results of Luo and co-workers and Ciannoccaro and co-workers, who suggested that autoantibodies against OX_2_ are not a typical feature in NT1 patients or in post-Pandemrix autoimmune responses [29,30]. Polyclonal commercially available rabbit polyclonal anti-OX_2_ immunoglobulins very specifically recognized OX_2_ in transiently transduced and transfected HuH7 cells, indicating that our techniques would be specific enough to detect OX_2_ autoantibodies in human sera if they had existed. In addition, analysis of anti-NP antibodies in patient sera with IFA and EIA readily showed antibody responses against influenza A virus NP and clearly detectable colocalization of human (patients sera) and rabbit anti-NP antibody staining in confocal microscopy. We also found no autoimmunity against the full-length prepro-orexin.

In a limited number of patient PBMCs, we could not demonstrate any cell-mediated immunity against the N-terminus of OX_2_. There is recent evidence that narcoleptic patients show cell-mediated immunity against Tribbles homolog 2 and especially against the orexin molecules [51]. However, in that study potential autoimmunity against orexin receptors was not analyzed.

## 5 Conclusions

In the present study, we found no evidence of humoral immunity against human OX_1_ or OX_2_ orexin receptors or the prepro-orexin molecule in a relatively large collection of serum specimens obtained from narcolepsy type 1 patients whose disease onset was associated with the use of Pandemrix vaccine. Thus, we found no evidence that influenza A virus vaccine antigen would induce cross-reactive antibodies between influenza virus nucleoprotein and orexin receptors in spite of putative molecular mimicry between viral NP and human OX_2_ receptor.

## Declaration of competing interest

The authors declare that they have no competing interests.
